# T-Cell Immunity in COVID-19-Recovered Individuals and Individuals Vaccinated with the Combined Vector Vaccine Gam-COVID-Vac

**DOI:** 10.3390/ijms24031930

**Published:** 2023-01-18

**Authors:** Sergey Petrovich Krechetov, Valentina Valentinovna Vtorushina, Evgenia Vladimirovna Inviyaeva, Elena Aleksandrovna Gorodnova, Svetlana Vladimirovna Kolesnik, Dmitry Anatolievich Kudlay, Pavel Igorevich Borovikov, Liubov Valentinovna Krechetova, Nataliya Vitalievna Dolgushina, Gennady Tikhonovich Sukhikh

**Affiliations:** 1 National Medical Research Center for Obstetrics, Gynecology and Perinatology Named after Academician V.I., Kulakov of the Ministry of Healthcare of Russian Federation, 117997 Moscow, Russia; 2 NRC Institute of Immunology FMBA of Russia, 115522 Moscow, Russia; 3 Department of Pharmacology, I.M. Sechenov First Moscow State Medical University (Sechenov University), 119991 Moscow, Russia; 4 Department of Obstetrics, Gynecology, Perinatology and Reproductology, I.M. Sechenov First Moscow State Medical University (Sechenov University), 119991 Moscow, Russia

**Keywords:** SARS-CoV-2, COVID-19, molecular pathology, infection, vaccination, Gam-COVID-Vac, humoral immunity, cellular immunity, cytokines, ELISPOT

## Abstract

The COVID-19 pandemic has required extensive research on the new coronavirus SARS-CoV-2 and the creation of new highly effective vaccines. The presence of T-cells in the body that respond to virus antigens suggests adequate antiviral immunity. We investigated T-cell immunity in individuals who recovered from mild and moderate COVID-19 and in individuals vaccinated with the Gam-COVID-Vac combined vector vaccine. The ELISPOT method was used to determine the number of T-cells responding with IFN-γ synthesis to stimulation by peptides containing epitopes of the S-protein or N-, M-, ORF3, and ORF7 proteins, using peripheral blood mononuclear cells (PBMCs). At the same time, the multiplex method was used to determine the accumulation of IFN-γ and other cytokines in the culture medium. According to the data obtained, the proportion of positive conclusions about the T-cell immune response to SARS-CoV-2 antigens in control, recovered, and vaccinated individuals was 12%, 70%, and 52%, respectively. At the same time, more than half of the vaccinated individuals with a T-cell response were sensitized to the antigens of N-, M-, ORF3, and ORF7 proteins not produced by Gam-COVID-Vac, indicating a high likelihood of asymptomatic SARS-CoV-2 infection. Increased IFN-γ release by single sensitized T-cells in response to specific stimulation in recovered and vaccinated individuals did not result in the accumulation of this and other cytokines in the culture medium. These findings suggest a balance between cytokine production and utilization by immunocompetent cells as a prerequisite for providing a controlled cytokine signal and avoiding a “cytokine storm”.

## 1. Introduction

In March 2020, the World Health Organization (WHO) classified the spread of the disease associated with SARS-CoV-2 virus infection as a COVID-19 pandemic [1]. Because of this decision, research on the SARS-CoV-2 coronavirus and the disease it causes, i.e., COVID-19, has been prioritized.

The intracellular parasitism of viruses significantly limits the body’s immune resistance to viral infection via antibodies [2,3,4]. The limited ability of humoral immunity to prevent virus infection of epithelial cells is exacerbated by the inability to maintain a high level of specific antibodies in the main entry gates of viral infection, i.e., mucous membranes and their surfaces [5,6,7]. However, when epitopes of synthesized viral proteins within the major histocompatibility complex (MHC) class I molecules are presented on the surface of infected cells [8,9], it becomes possible to detect and suppress infected cells via T-cell mechanisms of recognition and removal of cells inside which foreign or own modified proteins are synthesized [10,11,12]. Cells expressing foreign viral epitopes induce a T-cell immune response with the formation of memory cells [13,14,15] that help to both boost and improve T-cell immunity upon re-infection with the same pathogen [16,17]. Therefore, the presence of T-cells in the body that are specifically activated by virus epitopes indicates the presence of an essential component of antiviral immunity, which could result from either natural (past infection) [18,19] or artificial (vaccination) [20,21,22] contact the immune system has had with the virus or its components.

IFN-γ production is a distinguishing feature of cytotoxic T-cells and Th1 T-helpers, which are activated when they come into contact with cells on whose surface has MHC class I and II molecules that represent foreign epitopes [23,24]. As a result, the ability of lymphocytes to produce IFN-γ is being extensively researched for an enhanced understanding of the general basis of T-cell immunity [25,26]. Furthermore, it will be instrumental in assessing the role of T-cells in specific manifestations of T-cell immunity, such as antiviral immunity [27,28]. The number and characteristics of peripheral blood lymphocytes producing IFN-γ, as determined by specific viral antigen stimulation, serve as parameters of the T-cell component of the body’s immune response to a viral infection or a vaccine [29,30].

The characteristics of virus interaction with the host organism and the development of the host’s immune system response to a viral infection demonstrate the limitations of vaccines only containing virus protein antigens. The inability of such vaccines to provide intracellular synthesis of viral proteins, as well as the absence of viral protein epitope presentation in MHC class I, results in only a humoral immune response [31,32]. Live-attenuated viral vaccines [33], a non-replicating DNA vector with an inserted viral DNA fragment [34], plasmids with an embedded viral DNA fragment [35], and mRNA encoding viral proteins [36] do not have this disadvantage. At the same time, only the first of the listed vaccines can fully present virus antigens, while the rest are limited to one or several proteins or even epitopes [37]. However, stringent requirements for attenuated virus strains and their production disqualify attenuated viral vaccines, albeit traditional, as top candidates for accelerated development amid a pandemic emergency [38]. This resulted in the development and practical application of genetically engineered antiviral vaccines, with combined adenoviral vector vaccines playing an important role [39].

This paper focuses on the characteristics of IFN-γ producing T-cells in COVID-19-recovered individuals and individuals vaccinated with the Gam-COVID-Vac combined vector vaccine (recombinant adenovirus particles containing the S-protein gene of the SARS-CoV-2 virus) [40]. Comparative studies of the T-cell response of the immune system to the natural viral infection SARS-CoV-2 and to vaccination with a vector vaccine against this virus are necessary not only to find effective ways to prevent and treat COVID-19. The obtained data contribute to the development of modern ideas about the general mechanisms of interaction between viruses and the human immune system.

## 2. Results

### 2.1. Assessment of T-Cell Immunity

**Figure 1:**
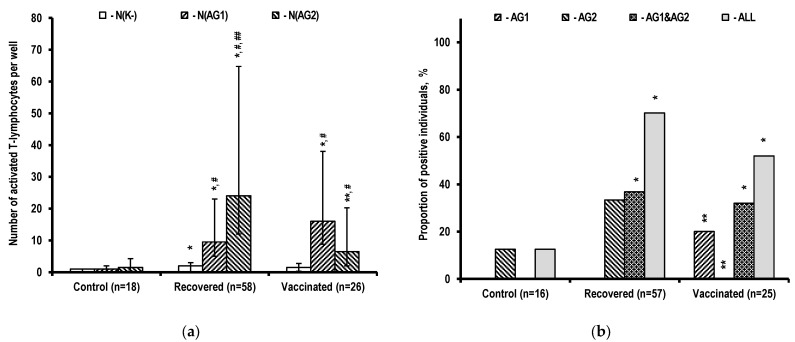
Activated T-cell count in PBMC samples. (**a**) Number of T-cells producing IFN-γ in wells without stimulation (N(K−)) and with specific stimulation using various sets of antigens (N(AG1), N(AG2)). The data are presented as Me (Q1; Q3). (**b**) Proportions of individuals testing positive for a T-cell immune response to SARS-CoV-2 virus antigens. AG1, AG2: Positive response to antigens of only one set. AG1&AG2: Positive response to antigens of both sets simultaneously. * *p* < 0.01 when compared to controls using the same parameter. ** *p* < 0.01 when compared to recovered individuals using the same parameter. # *p* < 0.01 when compared to non-stimulated PBMCs within a group. ## *p* < 0.01 when comparing PBMCs stimulated with various sets of antigens within a group.

The response of numerous cells to non-specific stimulation with OKT3 (murine monoclonal anti-CD3 antibody) leads to the formation of confluent spots from several cells and to faulty test results. The relevant data were therefore excluded from the analysis of the results of determining the number of activated T-cells producing IFN-γ (Figure 1). The results suggest that COVID-19 disease and Gam-COVID-Vac vaccination cause a significant increase in the responding T-cell count in the body. These cells respond to specific stimulation by both sets of peptides with increased IFN-γ production. Thus, recovered and vaccinated individuals have more spots in the stimulation wells, compared to controls (Figure 1a). T-cells activated by AG2 peptides (peptides with N-protein, M-protein, and ORF3 and ORF7 protein epitopes) predominate in recovered individuals. Vaccinated individuals with a predominance of T-cells activated by AG1 peptides (peptides with epitopes of the viral envelope spike S-protein) exhibit significant amounts of lymphocytes activated by AG2 peptides, whose epitopes should not have appeared in the body during vaccination.

The number of positive test results for a cellular immune response to SARS-CoV-2 virus antigens evaluated using the manufacturer’s criteria has no effect on the observed representation of T-cells that respond to different antigen compositions in a set in the study groups (Figure 1b). It should be noted that in this assessment for the composition of antigens that cause a reduced response, there are no individuals with a positive conclusion based solely on a significant response to these antigens in the respective groups. For example, there are no positives with a significant response only to AG1 antigens among the recovered individuals, and only to AG2 antigens among the vaccinated individuals. At the same time, at least half of the positives in these groups have both T-cells that respond to AG1 antigens and T-cells that respond to AG2 antigens. In total, the proportions of positive conclusions about the T-cell response to antigens in control, recovered, and vaccinated individuals were 12%, 70%, and 52%, respectively.

**Figure 2:**
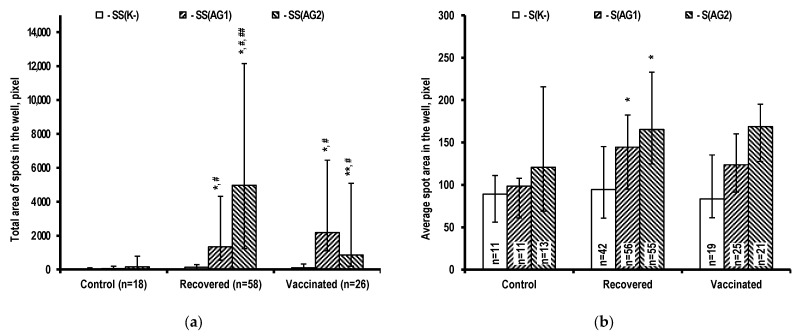
IFN-γ production intensity by PBMCs in the examined individuals. (**a**) Total spot area per well without stimulation (SS(K−)) and with specific stimulation using various sets of antigens (SS(AG1), SS(AG2)). (**b**) Mean spot area per well (wells without spots were excluded from analysis) without stimulation (S(K−)) and with specific stimulation using various sets of antigens (S(AG1), S(AG2)). * *p* < 0.01 when compared to controls using the same parameter. ** *p* < 0.01 when compared to recovered individuals using the same parameter. # *p* < 0.01 when compared to non-stimulated PBMCs within a group. ## *p* < 0.01 when comparing PBMCs stimulated with various sets of antigens within a group.

Using constant microscope magnification and image capture resolution, the area of the spot that forms around the cell at the bottom of the well is proportional to the amount of IFN-γ produced by that cell. Therefore, the total area of spots included in the analysis, as measured with ImageJ software using well images, is an estimate of the total amount of this cytokine produced by activated T-cells in the well. The results of measuring the total area of the spots included in the analysis using well images (Figure 2a) show that group features of the T-cell immune response in the individuals studied are similar to those observed when the number of responding PBMCs is counted (Figure 1a). These features include the predominant response of T-cells activated by AG2 antigens in recovered individuals, and the predominant response of T-cells activated by AG1 antigens, as well as a significant T-cell immune response to AG2 antigens not induced by the Gam-COVID-Vac vaccine, in vaccinated individuals. The observed similarity of the group features of the T-cell immune response in the examined individuals, as measured by the number of responding PBMCs and total IFN-γ production by these cells, is due to the greater number of responding lymphocytes for total cytokine production. Although higher, the parameters of IFN-γ production by individual sensitized T-cells following stimulation with viral antigens from various sets are quite close to that of activated T-cells in samples without stimulation; this increased value is significant only in recovered individuals (Figure 2b).

### 2.2. Determination of Antibodies Specific to SARS-CoV-2 Antigens

**Figure 3:**
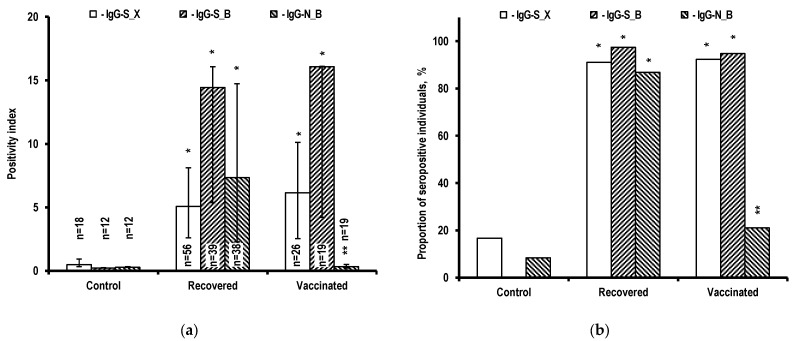
Results of the assay for serum detection of antibodies to SARS-CoV-2 protein antigens in the examined individuals. (**a**) Positivity indices (PIs) based on ELISA results. The data are presented as Me (Q1; Q3). (**b**) Proportions of seropositive individuals (PI > 1.1) in the examined groups, with regard to SARS-CoV-2 protein antigens. * *p* < 0.01 when compared to serum samples of controls using the same method. ** *p* < 0.01 when compared to serum samples of recovered individuals using the same method.

The results of the assay for serum detection of IgG antibodies to the S-protein receptor-binding domain (IgG-S_X), IgG antibodies to all S-protein epitopes (IgG-S_B), including the receptor-binding domain, and IgG antibodies to nucleocapsid N-protein (IgG-N_B) of the SARS-CoV-2 virus are shown in Figure 3.

According to the positivity indices obtained (Figure 3a), the antibodies of interest are poorly detected in control individuals, whereas recovered and vaccinated individuals show a significantly higher level of antibodies, corresponding to the antigen structure of the source that caused the humoral immune response. For example, the level of antibodies to the S-protein, as well as N-, M-, ORF3, and ORF7 proteins is significantly higher in recovered individuals whose immune systems interacted with a fully functional SARS-CoV-2 virus. At the same time, in vaccinated individuals with an artificial virus producing a fragment of the SARS-CoV-2 virus S-protein as the source of antigens, antibodies to this protein were detected at a high level, while antibodies to the N-protein were detected at a low level. The described features of seroconversion are preserved when seropositive individuals are identified using the ELISA criteria (Figure 3b). According to the obtained data, individuals seropositive for antibodies to S-protein antigens account for the majority (>90%) of recovered and vaccinated individuals, while individuals seropositive for antibodies to N-protein antigens account for approximately 90% of only the recovered group. In the vaccinated group, the proportion of individuals seropositive for antibodies to N-protein antigens is significantly lower than in the recovered group and corresponds to the level of control individuals with low seropositivity for antibodies to S-protein.

### 2.3. Cytokine Content in the PBMC Culture Medium

**Figure 4:**
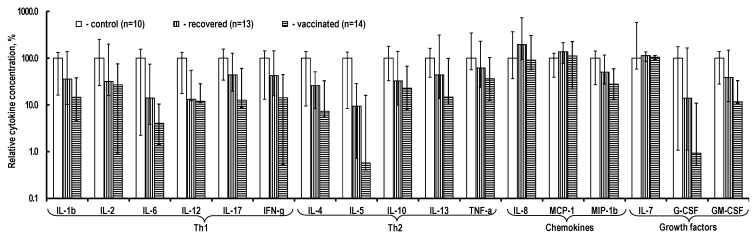
Cytokine content in the PBMC culture medium without stimulation. The relative cytokine concentration is the ratio of the measured absolute concentration of a cytokine in a sample to the median absolute concentration of this cytokine in control individuals’ samples. The data are presented as Me (Q1; Q3).

In the absence of stimulation (Figure 4), of note is the systematic but unreliably reduced content of almost all cytokines in the PBMC culture medium in recovered and vaccinated individuals. This characteristic is more prominent in vaccinated individuals. These trends are absent only for chemokines IL-8 and MCP-1, as well as growth factor IL-7.

**Figure 5:**
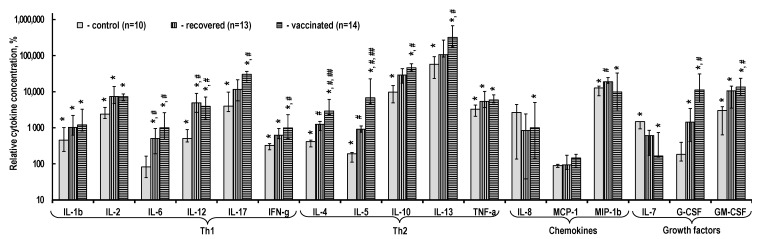
Cytokine content in the PBMC culture medium with non-specific stimulation by OKT3. The relative cytokine concentration is the ratio of the measured absolute concentration of a cytokine in a sample to the median absolute concentration of this cytokine in samples without PBMC stimulation in this group of individuals. * *p* < 0.01 when comparing samples without stimulation to samples with non-specific stimulation within a group. # *p* < 0.01 when comparing samples with non-specific stimulation between the group of interest and control group. ## *p* < 0.01 when comparing samples with non-specific stimulation between the groups of vaccinated and recovered individuals. The data are presented as Me (Q1; Q3).

Non-specific polyclonal stimulation with OKT3, monoclonal antibodies to the surface marker CD3 of T-cells, results in a significant increase in the content of most cytokines in the culture medium with PBMCs from all groups (Figure 5). At the same time, a greater increase in the content of cytokines in the medium with non-specifically stimulated PBMCs, compared to non-stimulated PBMCs, is observed in both recovered and vaccinated individuals. While the response to non-specific stimulation of most cytokines corresponded to the described characteristics, the shift in cytokine content in the culture medium with non-specifically stimulated PBMCs for the above-mentioned chemokines IL-8 and MCP-1 and growth factor IL-7 was unreliable and inconsistent with the trend of a greater increase in cytokine content in recovered and vaccinated individuals.

**Figure 6:**
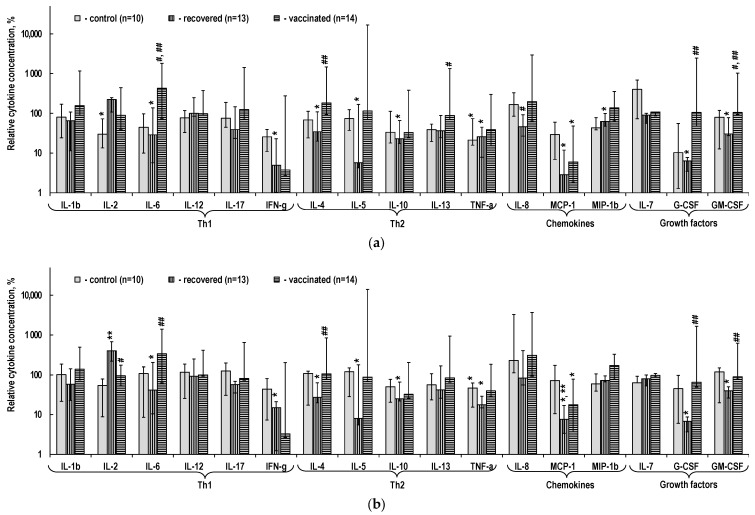
Cytokine content in the PBMC culture medium with specific stimulation by SARS-CoV-2 peptides. (**a**) AG1 peptides stimulation. (**b**) AG2 peptides stimulation. The relative cytokine concentration is the ratio of the measured absolute concentration of a cytokine in a sample to the median absolute concentration of this cytokine in samples without PBMC stimulation in this group. * *p* < 0.01 when comparing samples without stimulation to samples with non-specific stimulation within a group. ** *p* < 0.01 when comparing samples with specific stimulation using various sets of SARS-CoV-2 peptides within a group. # *p* < 0.01 when comparing samples with non-specific stimulation between the group of interest and control group. ## *p* < 0.01 when comparing samples with similar non-specific stimulation between the groups of vaccinated and recovered individuals. The data are presented as Me (Q1; Q3).

Unlike non-specific stimulation with OKT3, specific stimulation with SARS-CoV-2 virus peptides in the form of AG1 (Figure 6a) and AG2 (Figure 6b) sets does not result in an increase in cytokine content in the PBMC culture medium in all groups. All instances of increased cytokine content in culture medium in the presence of virus peptides compared to non-stimulated PBMCs are unreliable. That being said, the content of cytokines is lower in the vast majority of cases, and significantly lower in many cases, in the presence of virus peptides. The inconsistency with general trends for IL-8 and IL-7 observed in the analysis of cytokine content in the PBMC culture medium without stimulation and with non-specific stimulation persists with specific stimulation, but is less pronounced. At the same time, the features of MCP-1 accumulation in the PBMC culture medium correspond to the main features described above for specific stimulation.

### 2.4. Bioinformatics Analysis

#### 2.4.1. Clustering of Individuals

**Figure 7:**
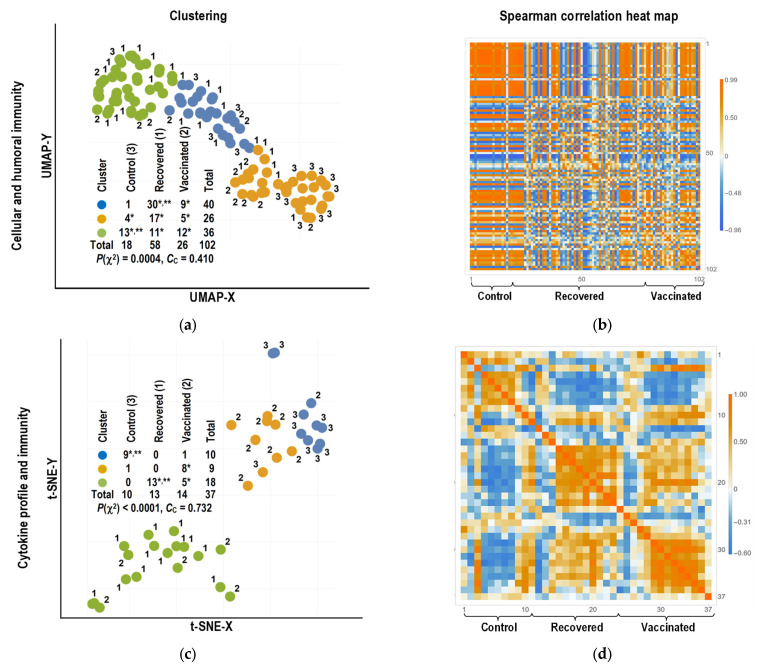
Bioinformatics analysis with individual clustering by cellular and humoral immunity parameters and cytokine profile of individuals. (**a**,**c**) Clustering results; (**b**,**d**) Spearman rank correlation coefficient heat diagrams. In images with clustering results, circles of the same color indicate individuals of the same cluster. The numbers near the circles represent the clinical group: 1—recovered individuals, 2—vaccinated individuals, and 3—control individuals. The presented contingency tables contain the number of representatives of each clinical group in the clusters. * The proportion of individuals in the cluster significantly exceeds 0.05 of the number of individuals with the corresponding clinical status (*p* < 0.01). ** The proportion of individuals in the cluster significantly exceeds the proportion of individuals with the corresponding clinical status in all other clusters (*p* < 0.01).

Bioinformatics analysis with individual clustering revealed that each of the three clusters (the number of clusters was chosen based on the number of study groups) identified by the algorithm with better clustering includes representatives from all groups, when using only the cellular and humoral immunity parameters measured in this study (Figure 7a). Nonetheless, despite the inability to accept the hypothesis of cluster composition coincidence due to the presence of individuals from different clinical groups (*p*(χ^2^) = 0.0004 and *C*_C_ = 0.410 for the corresponding contingency table in insertion), the significant differences obtained do not allow us to characterize any cluster as formed by representatives of any clinical group (absence of areas with high rank correlation in the heat diagram Figure 7b). For example, there is a significant number of recovered (11/58) and vaccinated (12/26) individuals in the cluster that includes most controls (13/18). Another cluster that includes the majority (30/58) of recovered individuals also contains a significant number of vaccinated individuals (9/26). The third cluster includes representatives of different clinical groups in significant numbers from 20 to 30% of the number of individuals in the groups.

When individuals are split into three clusters based on the parameters of cellular and humoral immunity as well as cytokine profile parameters (Figure 7c), the clusters are distinguished both by improved contingency of the representation table of individuals from different clinical groups (*p*(χ^2^) < 0.0001 and *C*_C_ = 0.732) and by improved linkage to clinical groups (presence of corresponding areas with high rank correlation on the heat diagram Figure 7d). There are no recovered individuals in the cluster that includes almost all the control subjects (9/10), and the proportion of vaccinated individuals is insignificant (1/14). All recovered individuals are part of another cluster, which also includes a significant number of vaccinated individuals (5/14), as was the case when cellular and humoral immunity parameters were used for clustering. The third cluster consists almost entirely of vaccinated individuals. It has no recovered individuals, and the number of controls is insignificant (1/10).

#### 2.4.2. Clustering of Parameters

**Figure 8:**
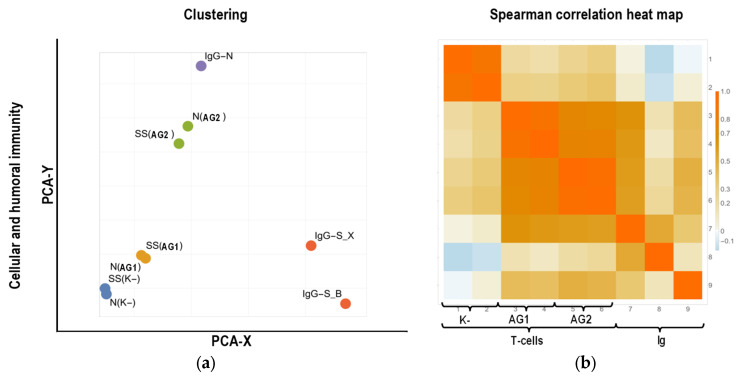

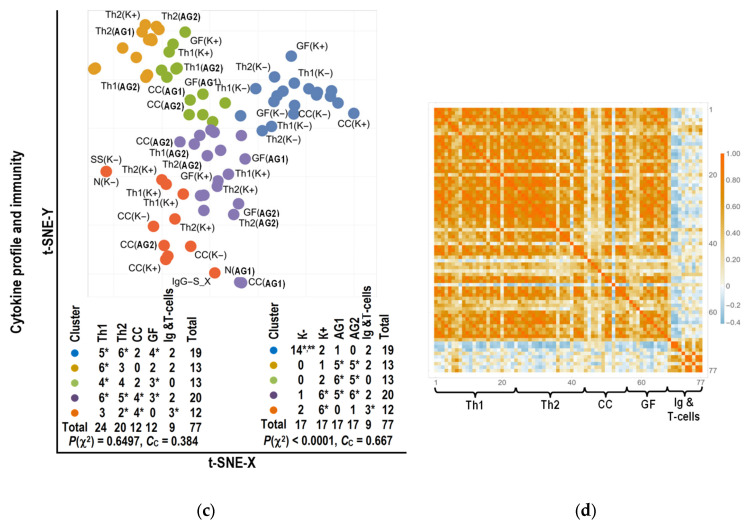
Bioinformatics analysis with clustering of cellular (T-cells) and humoral (Ig) immunity parameters and cytokine profile of individuals. (**a**,**c**) Clustering results; (**b**,**d**) Spearman rank correlation coefficient heat diagrams. In images with clustering results, circles of the same color indicate parameters of the same cluster. The designations near the circles represent the group of cytokines (Th1, Th2, CC, GF), cellular (N, SS) and humoral (IgG-S, IgG-N) immunity; the activation used (K+, AG1, AG2) or its absence (K−) is specified in brackets. The insertions in the lower clustering diagram show contingency tables with different groupings of indicators. * The proportion of parameters in the cluster significantly exceeds 0.05 of the number of parameters belonging to the specified group (*p* < 0.01). ** The proportion of parameters in the cluster significantly exceeds the proportion of parameters belonging to the specified group in other clusters (*p* < 0.01).

Clustering of parameters of the examined groups, limited only by cellular and humoral immunity parameters (Figure 8a,b), was linked to five clusters, based on the four specificities of evaluated antigen-recognizing structures (antibodies to S-protein (Ig-S_X, Ig-S_B), antibodies to N-protein (Ig-S_N), T-cells to S-protein peptides (AG1), or T-cells to N-, M-, ORF3-, and ORF7 protein peptides (AG2)), and parameters of non-stimulated lymphocytes (N(K−), SS(K−)). In this case, individual clusters combined the parameters assessed by specific antigen-recognizing structures and non-stimulated lymphocyte parameters.

Clustering of parameters of individuals, including cytokine production by lymphocytes (Figure 8c,d), was also linked to five clusters. Four clusters are required to assess the relationship between clustering results and cytokine grouping according to functional orientation (Th1, Th2, CC, GF) or lymphocyte activation (K−, K+, AG1, AG2). The addition of the fifth cluster was prompted by the need to assess the potential specific behavior of cellular and humoral immunity parameters (NN, SS, and Ig). Statistical evaluation of contingency tables shows the absence of significant differences between clusters (*p*(χ^2^) = 0.6497) when analyzing the representation of parameters, factoring in the functional orientation of cytokines. However, when analyzing cytokine parameters, accounting for lymphocyte activation, the differences between clusters become significant (*p*(χ^2^) < 0.0001, *C*_C_ = 0.667), with one cluster containing almost all (14/17) parameters of cytokine production by lymphocytes without activation. At the same time, no single cluster can be distinguished by a significant predominance of cytokine production parameters during lymphocyte activation, as well as cellular and humoral immunity parameters.

## 3. Discussion

### 3.1. Specific T-Cell Immune Response Characteristics during SARS-CoV-2 Infection and after Vaccination

The observed representation of the specificities of T-cells producing IFN-γ upon antigen stimulation of PBMCs in culture (Figure 1) after COVID-19 corresponds to the presentation in the MHC complexes of the epitopes of all proteins of the SARS-CoV-2 virus developing inside the cells of the body and reflects a higher level of T-cell response to antigens of proteins included in the AG2 set, specifically, the N-protein [41] or ORF1 and ORF3 proteins [42]. At the same time, in Gam-COVID-Vac vaccination, proteins with AG2 epitopes are not produced by the recombinant adenoviral particles used, which only contain the virus S-protein gene. The presence of T-cells responding to AG2 antigens in this case could be explained by unnoticed asymptomatic SARS-CoV-2 infection [43], which can occur before, after, and during vaccination.

Failure to characterize T-cell immune response as positive in a significant proportion of recovered (30%) and vaccinated (approximately 50%) individuals suggests a high probability of an undetected weak T-cell immune response when infected with the SARS-CoV-2 virus, without clinical manifestations of COVID-19. Although the number of positive T-cell responses in the control group was not significant, it is worth noting that AG2 antigens elicited a positive response in this group. This group is similar to the recovered group in this regard, and it suggests the possibility of developing a weak T-cell immune response during asymptomatic infection with the SARS-CoV-2 virus, which can only be detected using extremely sensitive methods [44,45].

The lack of significant intragroup differences in IFN-γ production by individual PBMCs without stimulation and with stimulation by viral antigens (Figure 2b) can be interpreted as a sign of a weak dependence of lymphocyte response to stimuli on antigen type and specificity of antigen-recognizing molecule interaction with it. When the activating signal caused by the interaction of the antigen with the recognizing specific molecular receptors of lymphocytes reaches a threshold level, cytokine synthesis begins [46,47], with the intensity determined by the biochemical capabilities of the body’s cells [48,49]. The metabolic characteristics of stimulated cells, which they acquire while maturing during primary antigen stimulation and the transition to memory cells (infection or vaccination) may be more important. The latter is confirmed by the significant difference in the area of the spots around the cells producing IFN-γ in the control and recovered groups, with stimulation by antigens (Figure 2b).

In contrast to T-cell immunity, a high level of antibodies in recovered and vaccinated individuals fully corresponds to the antigen composition of the antigen source that elicited the humoral immune response (Figure 3). There was a significant increase in the level of antibodies to both the S-protein and the N-protein in recovered individuals whose immune system interacted with a fully functional SARS-CoV-2 virus. At the same time, in vaccinated individuals whose antigen source was an artificial virus, a high level was found only for antibodies to the S-protein. For antibodies to N-protein antigens, the proportion of seropositive vaccinated individuals is comparable to that of control individuals.

### 3.2. Cytokine Production by T-Cells Sensitized upon Infection with SARS-CoV-2 and after Vaccination

The results for non-stimulated and stimulated cytokine profiles in the PBMC culture medium in this study are consistent with the fact that cytokine content in the culture medium is determined by both cytokine production by activated cells and its removal from the medium as a result of absorption by cultured cells, receptor and non-specific binding to the cell surface, and destruction in the culture medium [50,51]. In this regard, the observed lack of growth and even a tendency to decrease in cytokine content in the PBMC culture medium after specific stimulation with SARS-CoV-2 virus peptides appears to reflect a peculiarity of responding T-cells, which is that their ability to absorb cytokines increases at the same rate as their ability to produce cytokines. This is evident in the case of IFN-γ, which demonstrated an increase in the number of T-cells that respond to stimulation by AG1 and AG2 peptides with increased production of this cytokine in both recovered and vaccinated individuals (Figure 1a). However, as total IFN-γ production by individual cells increases, the content of IFN-γ in the culture medium of specifically stimulated PBMCs falls even lower than in the medium of non-stimulated PBMCs (Figure 6). The outflow of IFN-γ from a single lymphocyte due to binding to the membrane at the bottom of the well, as well as the total outflow from the medium due to absorption by non-responding PBMCs in the well, should be unchanged. Thus, the lack of an increase in this cytokine content in the culture medium of stimulated PBMCs could be attributed to an increase in cytokine uptake by T-cells that responded to stimulation.

The conclusion about the simultaneous increase in the intensity of both cytokine production and absorption by activated cells corresponds to the need for a controlled cytokine response as a condition for preventing hypercytokinemia and “cytokine storm” [52,53]. The statement about an increase in the uptake of cytokines by sensitized T-cells during specific stimulation is supplemented by the data obtained in the absence of stimulation (Figure 4), when an unreliable, but systematically reduced content of most cytokines is observed in the c culture medium of recovered and vaccinated individuals.

These findings could imply that a more intense uptake of cytokines by sensitized T-cells after an intense antigen load occurs not only in activated cells, but also in non-activated cells. However, the data suggest that the response to non-specific stimulation is accompanied by a predominance of cytokine production over their uptake by all PBMCs in culture (Figure 5). On the one hand, this could be due to peculiar molecular rearrangements of the T-cell receptor, which underpin the activation of non-specific T-cell signaling pathways [54,55] that differ from those activated by specific stimulation [56]. On the other hand, this could be associated with the presence of a large number of immature naive T-cells among PBMCs [57,58], which respond differently to non-specific stimulation than mature T-cells, possibly with a preference for cytokine production over their absorption.

### 3.3. Bioinformatics Analysis

When clustering individuals by cellular and humoral immunity parameters (Figure 7a,b), the low contingency of cluster composition with clinical characteristics of groups obtained in this study is consistent with the presence of a recombinant mechanism for the development of specific antigen-recognizing molecular structures (receptors) of naive T and B cells [59,60,61]. This mechanism scatters the intraindividual affinity of antigen-recognizing molecules for different epitopes [62], causes different affinities for the same epitopes in different individuals [63,64], determines the presence of polyclonal T and B memory cells in one individual [65,66], and also leads to differences in polyclonality and antigen-responsive cell count in different individuals [67,68]. All of the above result in the mutual independence of the distributions of specific cellular and humoral immune response parameters in recovered and vaccinated individuals, as well as the presence of a relatively large number of individuals with low immune response rates in these groups. As a result, there is a significant presence of representatives from various immunostimulated groups in all clusters, including the cluster with the greatest number of control individuals.

The stronger association with clinical groups in clusters derived from individuals’ cytokine profiles (Figure 7c,d) is consistent with the fact that cytokine production by lymphocytes responding to stimuli mediated by antigen-recognizing molecules is more dependent on stimulated cells’ metabolic characteristics, which they acquire during maturation when infected or vaccinated [48,49]. As a result, a cluster made up almost entirely of control individuals (without primary antigen stimulation), a cluster made up almost entirely of vaccinated individuals, and a cluster made up of all recovered individuals and a significant number of vaccinated individuals can be reliably distinguished.

When clustering the parameters of individuals (Figure 8a,b), limiting only cellular and humoral immunity parameters results in the expected cluster composition. One cluster’s parameters characterize either the same property of the individual (the content of antibodies to the S-protein) or properties that are strongly related to it (the number of cells producing IFN-γ and the total amount of IFN-γ produced by these cells). Clustering of parameters with the inclusion of an individual’s cytokine profile in the analysis does not reveal a link between the selected clusters and groups of cytokines (Figure 8c,d). The result obtained may reflect the mismatch and inconsistency in the pathways for the perception and transmission of control signals that trigger the production of individual cytokines in the cell [69,70], resulting in inconsistency in the production of different cytokines even within the same group.

However, when cytokine parameters are grouped according to the presence and type of stimulation of their production by PBMCs in culture, the association between non-stimulated cytokine production and the general level of biosynthesis in the individual’s cells is observed, as discussed in the analysis of the results of individual clustering using cytokine parameters (Figure 7c,d). As a result, most of the parameters of non-stimulated production of cytokines by PBMCs are concentrated in one cluster, with only a minor presence of stimulated production parameters. At the same time, none of the parameters of stimulated cytokine production are distributed systematically among the remaining clusters. The latter illustrates the previously mentioned discordance between the signaling metabolic pathways responsible for control signal perception and transmission, as well as the subsequent synthesis of various cytokines [71,72,73].

In general, the results show that the recombinant mechanism of antigen-recognizing T- and B-cell receptor specificity during the formation of adaptive antiviral immunity leads to different combinations of the body immune system’s ability to a cellular and humoral response. In most cases, the resulting combinations provide the body’s resistance to viral infection. However, in some cases, the resulting combination of immune response components is discordant [43]. This can result in the body’s emerging antiviral immunity being unable to completely rid itself of re-infection with the virus and its negative consequences [74], particularly the severe course of the disease in vaccinated individuals and re-infection in recovered individuals [75,76]. In this regard, it is critical for medical counteraction to infectious diseases caused by SARS-CoV-2 and other respiratory viruses to create conditions for full-fledged medical and equipment support for the treatment of patients with symptoms necessitating a hospitalization [77].

## 4. Materials and Methods

### 4.1. Samples

The study was carried out at National Medical Research Center for Obstetrics, Gynecology and Perinatology, named after Academician V.I. Kulakov of the Ministry of Healthcare of the Russian Federation, Moscow, Russia (hereinafter the Center). The Center has a high potential for COVID-19-related research based on an extensive biobank. The biobank premises comply with international GLP recommendations, and are equipped with laminar flow hoods, refrigerated centrifuges, and refrigeration equipment. All collections include various types of biomaterials for genetic, biochemical, immunological, and omics studies.

The study used venous blood samples taken from COVID-19-recovered individuals, individuals vaccinated against COVID-19, and those unvaccinated against COVID-19 who did not have a positive PCR test for SARS-CoV-2. The biomaterial was obtained from the Center’s biobank immediately after it was collected from the individuals included in the study. BD Vacutainer tubes (Becton Dickinson, NJ, USA) with sodium citrate were used to collect 8 mL of peripheral venous blood from the cubital vein for isolation of peripheral blood mononuclear cells (PBMCs), and another 1 mL of the same blood sample was collected into dry BD Vacutainer tubes to obtain serum. PBMCs were isolated by the conventional method [78] in the density gradient (*ρ* = 1.077 g/cm^3^) based on Ficoll solution (NPP PanEco, Moscow, Russia). Serum was separated by centrifugation at 1000g for 20 min to precipitate a clot of blood coagulation system components formed during a 30-min incubation of whole blood at room temperature.

### 4.2. Patients

The experimental study included 102 blood samples. The general inclusion criteria were as follows:

- signed informed consent;
- age ≥ 18 years;
- negative PCR test during blood sampling for the study;
- absence of concomitant diseases and conditions associated with impaired immunity (autoimmune diseases, malignant neoplasms, HIV infection);
- no treatment with immunomodulatory drugs within three months prior to participation in the study.

The group of recovered individuals (“recovered”) included blood samples taken from individuals with a clinical diagnosis of COVID-19 in the catamnesis, confirmed by a positive PCR test for SARS-CoV-2. The study included individuals with a history of a mild or moderate disease that did not require intensive care in a specialized inpatient setting.

The severity of COVID-19 was established based on the interim guidelines of the Ministry of Health of the Russian Federation “Prevention, diagnosis and treatment of a new coronavirus infection (COVID-19)” [79].

Mild COVID-19:

- body temperature < 38 °C, cough, fatigue, sore throat;
- absence of criteria for moderate and severe COVID-19.

Moderate COVID-19:

- body temperature > 38 °C;
- respiratory rate > 22 breaths per minute;
- shortness of breath during physical exertion;
- changes in CT (radiography), typical of a viral lesion;
- SpO2 < 95%;
- serum CRP > 10 mg/L.

The group of vaccinated individuals (“vaccinated”) included blood samples taken from individuals fully vaccinated against SARS-CoV-2 using a two-shot combined vector vaccine Gam-COVID-Vac (Gamaleya Research Institute of Epidemiology and Microbiology, Russia), which are recombinant adenoviral particles containing the S-protein gene of this virus. The vaccine components comprise two different vectors, recombinant adenovirus type 26 (first dose) and recombinant adenovirus type 5 (second dose), which carry the gene for SARS-CoV-2 full-length glycoprotein S. A dose of viral particles is 10^11^ for each recombinant adenovirus. The vaccine preparation is a liquid and is stored frozen. After thawing, the 0.5 mL vaccine components are injected intramuscularly into the deltoid muscle at 21-day interval following the manufacturer’s recommendations. The vaccine induces humoral and cellular immunity against the SARS-CoV-2 virus [40,80].

The control group (“control”) included blood samples taken from unvaccinated individuals with no history of COVID-19.

Blood samples of recovered individuals were collected at least 30 days after the previous positive PCR test for SARS-CoV-2. Blood samples were collected from vaccinated individuals at least 30 days after the administration of the second component of the vaccine, and from controls immediately after a negative PCR test.

Biomaterial of 102 individuals from the biobank collections was selected for the study. The control, recovered, and vaccinated groups included 18, 58, and 26 individuals, respectively. A total of 71 women (69.6%) and 31 men (30.4%) were included in the study. The mean age of the study participants, irrespective of the group, was 49.0 (39.5; 54.8) years. The characteristics of the individuals included in the study, by groups, are presented in Table 1. There were no significant differences between the groups in terms of gender and age.

Characteristics of COVID-19-recovered individuals, following the recommendations of the Ministry of Health of the Russian Federation [79], are summarized in Table 2. Mild COVID-19 was distinguished by a subfebrile temperature and the absence of signs of lung damage and other signs of severe and moderate infection. Moderate disease was distinguished by a temperature above subfebrile (38.0 °C) and lung damage of no more than 50%, evidenced by a CAT scan. The clinical manifestations of the disease at varying degrees of severity included olfaction reduction/anosmia, fever, headache, general weakness, myalgia, and cough. The duration of clinical manifestations of the disease in the examined individuals ranged from 3 to 30 days.

Vaccination of individuals included in the study was not associated with serious post-vaccination complications, and the prevalence of complications in men and women was comparable (Table 3). The complications after the administration of the vaccine components were short-term (1–2 days), and their duration did not differ significantly between the components.

### 4.3. Virus Identification

Virus identification was carried out in oropharyngeal swabs obtained using disposable probes (Alife-Dafina LLC, Moscow, Russia). RNA was isolated using the PROBA-NK reagent kit (NPO DNA-Technology LLC, Russia). Amplification was carried out using a DT-964 instrument (NPO DNA-Technology LLC, Moscow, Russia). The results were processed automatically using the equipment software.

### 4.4. Assessment of T-Cell Immune Response

The T-cell immune response was assessed using the TigraTest SARS-CoV-2 reagent kit (JSC GENERIUM, Moscow, Russia), designed to count T-cells secreting gamma-interferon (IFN-γ). The method is based on the formation of enzyme-linked immunospots (ELISPOT, Enzyme Linked SPOT analysis) on a membrane with ligated antibodies to IFN-γ around T-cells secreting IFN-γ (Interferon Gamma Release Assay, IGRA). For specific stimulation, the test used two sets of peptides with epitopes of different SARS-CoV-2 virus proteins. One set (AG1) includes peptides with epitopes of the viral envelope spike S-protein, while the other set (AG2) includes peptides with epitopes of the nucleocapsid N-protein, membrane M-protein, and auxiliary (non-structural) proteins (ORF3, ORF7). The test was carried out following the manufacturer’s instructions. To process the test results, images of the bottom of the wells were obtained using a DTX 90 digital microscope (Levenhuk, Tampa, FL, USA) at a fixed magnification. Spots in the wells were identified and analyzed by electronic image processing using the ImageJ 153e software package (National Institutes of Health, Bethesda, MD, USA). The black-and-white images with the same resolution were analyzed for particles (Analyze Particles), with the number of particles and their areas in pixels determined. The analysis included spots with an area of at least the 90th percentile of the spot area distribution for all spots obtained in wells without stimulation with PBMC from control individuals. Per the manufacturer’s recommendations, the result of the test for the presence of a T-cell immune response to SARS-CoV-2 virus antigens was considered positive if the difference between the number of spots in the wells without stimulation and with PBMC stimulation by antigens from at least one set exceeded 12.

### 4.5. Determination of Antibody Content

Determination of the content of antibodies to SARS-CoV-2 proteins in serum was carried out by an enzyme-linked immunosorbent assay. We used reagent kits for the determination of IgG antibodies to the receptor-binding domain (RBD) of the S-protein “SARS-CoV-2-IgG-ELISA” (CHEMA LLC, Moscow, Russia) and IgG antibodies to S- and N-proteins of SARS- CoV-2 “SARS-CoV-2-AT spectrum-IFA-BEST” (Vector Best JSC, Novosibirsk, Russia). The study was carried out following the manufacturer’s instructions, with optical density measured on a microplate enzyme immunoassay analyzer Infinite F50 (Tecan, Grödig, Austria). The results were recorded by a positivity index (PI) calculated according to the manufacturer’s instructions, and the individual was considered seropositive if PI > 1.1.

### 4.6. Determination of Cytokine Concentration

The concentration of cytokines in the samples of the PBMC culture medium obtained during the T-cell immune response evaluation was determined by the multiplex method using the 17-plex Bio-Plex Pro Human Cytokine 17-plex Assay panel (Bio-Rad, Hercules, CA, USA). The content of cytokines of the Th1 (IL-1β, IL-2, IL-6, IL-12, IL-17, IFN-γ) and Th2 (IL-4, IL-5, IL-10, IL-13, TNF-α) groups, chemokines (IL-8, MCP-1, MIP-1β), and growth factors (IL-7, G-CSF, GM-CSF) was determined. A Bio-Plex 200 flow laser fluorimeter (Bio-Rad, Hercules, CA, USA) was used for measurements. The measurement results were processed using the Bio-Plex Manager 6.0 Properties application (Bio-Rad, Hercules, CA, USA).

### 4.7. Statistical Analysis

Statistical analysis of the obtained results was performed using the Microsoft Office Excel 2007 (Microsoft, Redmond, WA, USA)) software and the MedCalc^®^ Software version 14.8.1 (MedCalc, Ostend, Belgium) statistical software package. Quantitative parameters are presented as median (Me) and interquartile range (Q1; Q3). Nonparametric tests were used to compare quantitative parameters between groups: Mann–Whitney test for unpaired measurements and Wilcoxon signed rank test for paired measurements. Null hypotheses were dismissed and differences were considered significant at *p* < 0.01. The significance level of 0.01 was chosen, as it is less than the value of 0.0167 required when using the Bonferroni correction [81] with paired comparisons for three groups and a significance level of 0.05 when testing statistical hypotheses multiple times.

Bioinformatics analysis was performed using the WOLFRAM MATHEMATICA 13.0 (Wolfram, Champaign, IL, USA) software package. Before clustering, the values of parameters of individuals were centered and normalized to the standard deviation (autoscaling). For downweighting, principal component analysis (PCA), uniform manifold approximation and projection (UMAP), and t-distributed Stochastic Neighbor Embedding (t-SNE) methods were used. A method was chosen that results in improved clustering on the plane. Clustering was performed according to the KMedoids algorithm. The specified software package was used to calculate the Spearman rank correlation coefficient and build heat diagrams. The distribution of individuals and parameters by clusters was evaluated by analyzing the contingency tables (Pearson’s χ^2^ test and contingency coefficient (*C*_C_)) and frequencies (z-test) using the MedCalc software package.

## 5. Conclusions

The presentation of all antigens by a fully functional SARS-CoV-2 virus (disease) and only S-protein antigens by the Gam-COVID-Vac vector vaccine (vaccination) determines the features of antiviral cellular and humoral immune responses in a sensitized organism. However, the inability to rule out an undetected asymptomatic infection during a pandemic appears to be the reason for a significant T-cell response to non-S protein antigens in vaccinated individuals.

If the presence of a humoral immune response is confirmed in more than 90% of infected and vaccinated individuals, the presence of a T-cell response can be confirmed in approximately 70% of recovered and 50% of vaccinated individuals. Thus, the recombinant mechanism of the formation of specific antigen-recognizing molecular structures (receptors) of naive T and B cells in the body results in a different level of cellular and humoral antiviral immunity in an individual following infection with the SARS-CoV-2 virus or vaccination with Gam-COVID-Vac.

In contrast to non-specific polyclonal stimulation, increased production of IFN-γ by individual T-cells sensitized with viral antigens is not accompanied by an increase in the content of this and other cytokines in the culture medium when cytokine production is stimulated in vitro by antigen peptides. These findings suggest that the ability of T-cells responding to specific stimulation to utilize cytokines increases along with their ability to produce cytokines. As a result, conditions are created with a specific immune response to ensure a controlled level of cytokines and prevent a “cytokine storm”.

## Figures and Tables

**Table 1:** Characteristics of the groups.

Group of Individuals	Total Individuals	Number of Women, n (%)	Number of Men,n (%)	Age, Years *	Period of Examination from the Date of the Event, Days *
Control	18	11 (61.1)	7 (38.9)	48.0 (38.0; 55.5)	—
Recovered	58	40 (69.0)	18 (31.0)	49.0 (42.0; 57.0)	169 (109; 366) **
Vaccinated	26	20 (76.9)	6 (23.1)	49.0 (41.0; 57.0)	99 (46; 161) ***

* The data are presented as Me (Q1; Q3). ** In recovered individuals, the period from the first positive conclusion according to the PCR test results is indicated. *** In vaccinated individuals, the period after the administration of the second component of the vaccine is indicated (the second component of the vaccine was administered 21 days after the first).

**Table 2:** Clinical characteristics of the course of the disease in COVID-19-recovered individuals (n = 58).

Clinical Characteristics of the Disease	Characteristic Value
Olfaction reduction/anosmia, n (%)	46 (79.3)
Headache, n (%)	41 (70.7)
Myalgia, n (%)	26 (44.8)
Cough, n (%)	24 (41.3)
Lung damage according to CT *, n (%)	16 (27.6)
Weakness, n (%)	35 (60.3)
Duration of mild disease, days **	7 (5; 12.8)
Duration of moderate disease, days **	10 (8; 18.8)

* Lung damage ≤ 50%. ** The data are presented as Me (Q1; Q3).

**Table 3:** Characteristics of post-vaccination complications (n = 26).

Post-Vaccination Complications	Number of Individuals with Post-Vaccination Complications, n (%)	
	After the Administration of Component 1	After the Administration of Component 2
Local reactions *	3 (11.5)	5 (19.2)
Subfebrile fever (37.1–38.0 °C)	8 (30.8)	7 (26.9)
Febrile fever (38.1–39.0 °C)	1 (3.8)	1 (3.8)
Systemic reactions **	5 (19.2)	5 (19.2)

* Tenderness, edema, erythema. ** Feeling unwell, headache, myalgia.

## Data Availability

Data sharing not applicable.
